# Example Is the School of Mankind

**Published:** 1899-08

**Authors:** 


### Editorial.

### "EXAMPLE IS THE SCHOOL OF MANKIND."

In our last issue expression was given to views held in regard to the conventions, that at this period dominate the thought of the active minds in dentistry, and as these will have been relegated to history before this number reaches our readers, it may be well to call attention to the exceeding value of a good example.

The thought usually connected with this is trite, and a moral homily is not proposed at this writing, nor would it be read in these August days, when nature and man grow indifferent to every effort save that of mere living.

The close of the conventions causes the thought, naturally, to turn to the moral accountability of these bodies, and it is here a word should be said. These organizations are the leaders of thought in the profession. If they fail in this duty, then they cease to be of value and deserve to go out of existence. This responsibility is, it is feared, not regarded by the members as it should be, hence the want of respect felt for these organizations by the best-thinking minds. There is a large body of men, active in their local societies, who are rarely or never seen at national gatherings. When the question is asked, Why do you not take an active interest in these organizations? the answer is almost invariably an expression of lack of confidence in their value. Those who do take part know that this is an error of judgment; but the fact that this feeling exists everywhere indicates a weakness somewhere in these associations that should be sought and the remedy applied. The National Dental Association, and all the State and inter-State associations, need these men, for they constitute the real strength of the profession, and it is time some measures were taken to draw them into activity in the larger fold, where the greatest measure of wisdom is needed.

The causes that have led to this indifference are rarely personal, and yet this is, by no means, a limited factor in driving men away. Two years ago the writer tried to call the attention of the members of the American Dental Association to the needs of that organization, or the one that might succeed it. It was broadly stated that one of the worst evils in that body was in the character of membership, that of permanent and delegated members. This was not understood then, and the explanation of the meaning will not be attempted now, but it may be stated that permanent membership means a clique, and this leads to control, and control disgusts the delegate who for the first time enters it as a representative. The result is a personal antagonism never overcome and a perpetual loss to the higher work of dentistry.

The duty of large organizations is to be exemplars of the best in everything. In so far as they fall short of this are they a weakness and a drag upon progress.

The dental profession in the United States has made most satisfactory growth in theory and practice, but in what may be called the higher moral excellencies it has remained practically where it was a half-century ago. It has failed to cultivate the professional spirit, and the reason why this has not been done lies largely at the doors of the national organizations. From the first body organized to the last there has been developed a petty commercial spirit, antagonistic to true professional spirit, and, in fact, has practically destroyed it.

This commercial spirit is so strongly intrenched in the minds of the members that any attempt to overcome it is always met with decided opposition. What is meant by this commercial spirit? The two national organizations now existing are the National Dental Association and the Oral Section of the Medical National Association. These, with the branches of the former, are supposed to concentrate the leading thought in dentistry. They have good meetings and a variety of papers of variable quality. What becomes of these? The meeting has hardly begun—indeed, oftentimes long before—when the proceedings are put up for sale. This may seem harsh, but it represents a fact. The matter is not auctioned off to the highest bidder, or are estimates ordinarily asked for, but that journal is selected that has the largest backing commercially, under the supposition that this will offer the most favorable conditions. The excuse has been given that, "We can get the proceedings printed and bound without cost to the organization, and thus have money in the treasury to spend for scientific objects." This excuse might be a valid one if the money were ever spent for objects of a scientific value. There have been occasional spasmodic efforts in this direction, but the "value" of the results remains an uncertain quantity.

What is needed in all dental organizations is a high moral example. They should be above reproach. They should publish at their own expense all proceedings, or give them freely to representative journals. The dignified way is to publish independently and let the journals copy if they so desire.

The laxity in moral stamina in the leading organizations has had its legitimate effect in driving out almost entirely the professional spirit. The subordinate societies, following this example, sell their proceedings with all the coolness of the auctioneer. That this is not true of all, this journal has reason to know and to be thankful for, but what kind of professional morality is it that will dicker with trade and then call dentistry a profession? It is safe to assume that it will never deserve the name of profession until it learns to make sacrifices for knowledge. No high position was ever attained by the aspirant for honors who attempted to gain it by dubious methods. Organizations and individuals must alike be true, and in proportion as they are true to the higher ethical law will both develop to the same standard. "Example goes before precept," and the example of the good and true in organizations and in individuals is the corner-stone of the building we are expected to rear for the future, and we will be held accountable by generations to come if it be not perfectly laid.

